# Split hand/foot malformation genetics supports the chromosome 7 copy segregation mechanism for human limb development

**DOI:** 10.1098/rstb.2015.0415

**Published:** 2016-12-19

**Authors:** Amar J. S. Klar

**Affiliations:** Gene Regulation and Chromosome Biology Laboratory, National Cancer Institute, Center for Cancer Research, National Institutes of Health, Building 539, Room 154, Frederick, MD 21702-1201, USA

**Keywords:** limb development, human split hand/foot malformation, selective chromatid segregation mechanism, selective DNA strand segregation mechanism, asymmetric cell division mechanism, DNA chirality-based developmental mechanism

## Abstract

Genetic aberrations of several unlinked loci cause human congenital split hand/foot malformation (SHFM) development. Mutations of the *DLX5* (*distal-less*) transcription factor-encoding gene in chromosome 7 cause SHFM through haploinsufficiency, but the vast majority of cases result from heterozygous chromosomal aberrations of the region without mutating the *DLX5* gene. To resolve this paradox, we invoke a chromosomal epigenetic mechanism for limb development. It is composed of a monochromatid gene expression phenomenon that we discovered in two fission yeasts with the selective chromosome copy segregation phenomenon that we discovered in mouse cells. Accordingly, one daughter cell inherits both expressed *DLX5* copies while the other daughter inherits both epigenetically silenced ones from a single deterministic cell of the developing limb. Thus, differentiated daughter cells after further proliferation will correspondingly produce proximal/distal-limb tissues. Published results of a Chr. 7 translocation with a centromere-proximal breakpoint situated over 41 million bases away from the *DLX* locus, centromeric and *DLX5*-region inversions have satisfied key genetic and developmental biology predictions of the mechanism. Further genetic tests of the mechanism are proposed. We propose that the DNA double helical structure itself causes the development of sister cells' gene regulation asymmetry. We also argue against the conventionally invoked morphogen model of development.

This article is part of the themed issue ‘Provocative questions in left–right asymmetry’.

## 1. Background

Biological research is in crisis—Technology gives us the tools to analyze organisms at all scales, but we are drowning in a sea of data and thirsting for some theoretical framework with which to understand it [1].

**Figure 1.**
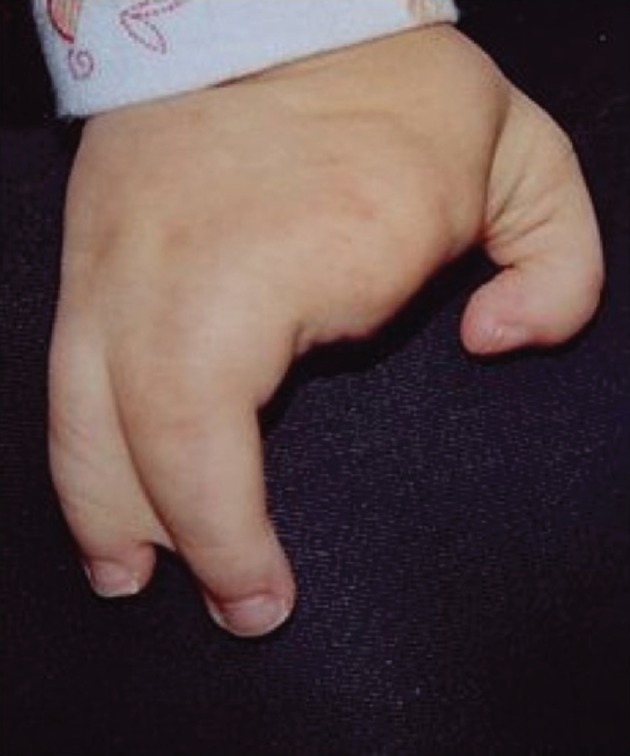
Split hand malformation (ectrodactyly, i.e. missing fingers) of the right hand (image from Wikipedia). The 4th and 5th fingers are partly united, an anomaly called syndactyly malformation.

Vertebrate limb development has been an active area of research in the field of developmental biology for many decades (reviewed in [2]). The morphogen gradient model [3] has been the primary paradigm followed for guiding research on limbs, as well as for the development of other organs during embryogenesis. However, despite decades of extensive research on development in all sorts of organisms, it is not understood precisely how developmental control genes are regulated, expressed or silenced at the correct position and with their exact timing in the course of development, nor how the development of different cells types and tissues is coordinated during embryogenesis. In the case of limb development, naturally arising limb developmental anomalies have provided a rich source of genetic material for discovering developmental mechanisms operating in humans. The congenital split hand/foot disorder (SHFM; online Mendelian inheritance in man (OMIM) 225300), also referred to as ectrodactyly (figure 1), is such a congenital defect in limb digit formation. SHFM consists of a spectrum of distal portion malformations of the hand/foot owing to a deep median cleft, missing digits and bones and missing digits and hypoplasia of the central rays [4]. The SHFM occurs as an isolated limb defect (non-syndromic and sporadic) or as part of a syndrome associated with defects of other organs, such as mental retardation, hearing loss and cleft lip and palate [5]. Also, the extent of the SHFM malformation phenotype is highly variable, and inexplicably, zero to four limbs can be malformed in the same genetically predisposed individual in familial cases.

As the relationship between SHFM genotype and phenotype is not understood, here, we present a new developmental biology framework for explaining the baffling genetics of chromosome (Chr.) 7 *SHFM1* locus aberrations affecting limb development without invoking mutations of the relevant *DLX5* gene as the cause of malformation. The *DLX5* gene encodes a transcription factor related to the *Drosophila distal-less* homeobox domain-containing protein, hence named *DLX* gene in humans. We exploit the genetics of Chr. 7 aberrations to dissect the molecular aetiology of the *SHFM1* locus and of the biology of limb development. Non-syndromic cases occur in about one in 18 000 births, and most of them are sporadic in nature. The aetiology of sporadic cases also remains unknown [6]; we propose the same chromosomal basis for their origin too. Our analysis aims to discover unknown aspects of the limb developmental mechanism. In essence, this study employs the model-based, hypothesis-testing approach of science to understand the biology of limb development. Here we argue that the complementarity of DNA strands itself can be converted into a/symmetric gene expression of daughter/sister cells, a phenomenon likely required as a key feature of eukaryotic development.

## 2. *DLX5* gene defines the *SHFM1* locus

Many of the genes and pathways for limb development have been remarkably conserved from *Drosophila* to humans [7]. SHFM is a heterogeneous condition caused by abnormalities of several loci in humans. At least seven loci have been identified for the familial inherited cases of SHFM through linkage or cytogenetic analyses [4,5,8–11]. These loci represent several chromosomal regions: *SHFM1* maps on Chr. 7q21 (MIM 183600); *SHFM2* on Chr. Xq26 (OMIM 313350); *SHFM3* on Chr. 10q24 (MIM 600095); *SHFM4* on Chr. 3q27 (MIM 605289) and *SHFM5* on Chr. 2q31. Homozygous *cathedrin-3* gene mutations cause SHFM, macular dystrophy syndrome and ectodermal dysplasia [12]. A p63 transcription factor, encoded by the *p63* gene comprising the *SHFM3* locus, binds to enhancers of the *DXL5/6* genes of the *SHFM1* locus to regulate gene expression during limb development [13]. p63 functions genetically upstream of the *DLX5* expression, and thus, the *DLX–p63* pathway defines the role of corresponding *SHFM1* and *SHFM4* loci in limb development [14]. These genes have been implicated in the development of the limb, craniofacial structures, the inner ear and the brain [15]. Importantly, the *DLX* gene functions in the Wnt signalling pathway required for limbs' skeletal development [10].

Relatively recently described intragenic *DLX5* gene mutations very clearly cause SHFM when in the heterozygous condition [11,16], but surprisingly, most cases are associated with heterozygous chromosomal aberrations of this 7q21 locus where the *DLX* gene itself is not interrupted or mutated [5,17–19]. These aberrations include deletions, translocations and inversions of the locus. Thus, a well-appreciated paradox persists in the literature whereby a gene mutation causes genetic disorder but paradoxically disorder is caused in most patients by chromosomal aberrations without the relevant gene having been mutated. Nearly all studies have hypothesized a position-effect control to explain this paradox such that the genomic rearrangements disrupt the normal expression pattern of the *SHFM1* locus *DLX5* gene by separating it from the required long-range-acting, *cis*-regulatory elements, resulting in decreased, increased or ectopic gene expression. Long-range gene regulators that function in developmental processes are well known. Surprisingly, such regulatory elements are dispersed in regions spread over hundreds of kilobases (Kb) upstream or downstream of the gene itself [20]. It is also well known that developmental control genes undergo tissue-specific and temporally express in time during development. However, the mechanisms for controlling the expression of *DLX* gene by position effects, tissue-specific expression and the biological basis of disorder-causing *SHFM1* locus chromosomal aberrations are not fully understood [20]. For the *SHFM1* locus-associated cases, an autosomal-dominant, incomplete penetrance and haploinsufficiency model for *DLX* gene regulation has been proposed in numerous studies [5,10]. Although this model helps to describe the preponderance of SHFM1 cases very well, it is not clear how to experimentally scrutinize its validity. Here in this hypothesis paper we propose a chromosomal epigenetic mechanism, a mechanism first discovered in studies of fission yeasts [21,22] and mouse cells [23,24], for differential regulation of the *DLX* gene of a specific pair of sister cells produced during limb development. In short, we aim to explain the enigmatic genetics of chromosomal aberrations that cause SHFM1 although their *DLX5* gene is not mutated.

## 3. The DNA *SSIS* produces sister cells of different cell types in two evolutionarily diverse fission yeasts

Pertinent to defining the developmental biology mechanisms at large, it is crucial to understand how developmentally equivalent or non-equivalent daughter cells are produced at specific stages of embryogenesis and during tissue homeostasis. Production of non-equivalent daughter cells is generally thought to be accomplished by regulated distribution of differentiation-specifying cellular factors and/or by differential exposure of daughter cells to cell-extrinsic factors [25]. Because different mechanisms might have evolved in different organisms, research with different organisms on this topic has continued for many decades. As a case in point, the mating-type switching phenomenon of fission yeasts has provided a powerful model system for understanding the biological mechanism of asymmetric cell division. In the highly diverged *Schizosaccharomyces pombe* [21,22] and *S. japonicus* [26] yeasts (reviewed in [27]), a DNA strand-specific, non-canonical imprint is installed at the mating-type locus (*mat1*) during its replication in mitotic cells. The imprint causes sister chromatids to differ by epigenetic means for their *mat1* gene activity. By this mechanism, only one of the two sister cells produces one daughter of switched mating/cell type, such that strictly one of four granddaughters of a cell switches in over 80% of cellular pedigrees. This unique mechanism of cellular differentiation is based fundamentally on the inherent DNA sequence differences in the *mat1* DNA strands. A key genetically predicted result that initially established the DNA strands asymmetry model was that yeast cells genetically engineered to carry an inverted *mat1* gene duplication produced equivalent sister cells because both sister cells became capable of producing switched progeny of their own [22]. That is, the usually non-equivalent sister cells became developmentally equivalent once the mother cell carries the inverted *mat1* duplication construct. The two chromosomal DNA strands carry DNA sequences that are complementary to one another, but they have opposite chemical polarities [28]. Furthermore, each strand serves as a template for synthesizing the complementary strand during chromosome duplication through the semi-conservative replication mechanism [29]. Thereby, each chromosome replication process produces paired daughter chromosome copies, which, in the G2 phase of the cell cycle, are called sister chromatids. Moreover, one chromatid always contains the arbitrarily designated Watson (W) DNA replication template strand and the newly synthesized Crick (C) strand, and the sister chromatid contains the template C strand and the newly synthesized W strand. Yeast sister chromatids are additionally differentiated by the inherent leading- versus lagging-strand mode of replication at the *mat1* locus [30,31]. To accomplish such precision, *mat1* is replicated strictly in a single chromosomal direction conducive for the imprinting process [32]*.* Altogether, the developmental asymmetry of daughter cells in fission yeasts is simply owing to the production and inheritance of the epigenetically differentiated *mat1* gene residing on sister chromatids. Mechanistically, installation of the epigenetic mark is based on the specific W versus C strand, older versus newly synthesized, and the leading- versus lagging-strand mode of DNA replication at the *mat1* locus. In short, the daughter cell's developmental asymmetry results from the parental cell's *mat1* replication history [21,22], and that is strictly based on the double-helix structure of DNA*.* The understanding of the DNA strand's chirality mechanism of cellular differentiation in fission yeasts has motivated us to propose the somatic strand-specific imprinting and selective sister chromatid segregation (SSIS) model as a mechanism for explaining the origin of vertebrates' visceral organs' left–right laterality [33,34] and of the human brain's hemispheric laterality development [35,36]. Here we advocate the same mechanism to explain both the precise regulation of *DLX* gene expression at a critical cell division during limb development and the aforementioned *SHFM1* locus chromosomal aberrations affecting limb development in humans.

## 4. The SSIS mechanism proposed for the *DLX5* gene regulation of a deterministic cell dividing in the limb bud anlagen

As stated above, correct spatial and temporal control of gene expression is essential for embryonic development, including limb development. Regulation of developmental control genes can be influenced by regulatory elements located some distance from the promoter regions, both in upstream and downstream regions of the gene [37], but their precise mechanism of regulation is not yet understood. Their transcriptional regulation is thought to be under chromosomal ‘position-effect’ controls. Many of these genes encode transcription factors with tissue- and stage-specific expression patterns. It is not known precisely how embryonic and stem cells establish a unique programme of gene expression that determines what kinds of daughter cells they will produce. Notably, the *DLX* gene encodes an evolutionarily conserved group of homeodomain transcription factors related to the *Drosophila distal-less* (*dll*) genes [14]. The *DLX* gene family consists of six gene clusters existing in vertebrates, which are generally organized into bi-gene clusters. In four-limbed (i.e. tetrapods) animals, the *DLX5* gene is expressed in the apical ectodermal ridge of the developing limb bud and it is clearly required for limb development because altered function of *DLX5* factor causes the human split hand/foot malformation 1 (SHFM1) development. The molecular signalling pathways involved in limb digit patterning and limb bud growth are best described by studies of the mouse and chicken model organisms [14]. In comparison, the mechanisms of how the *DLX5* gene for limb development is precisely expressed or silenced in different human limb tissues remain undefined.

**Figure 2.**
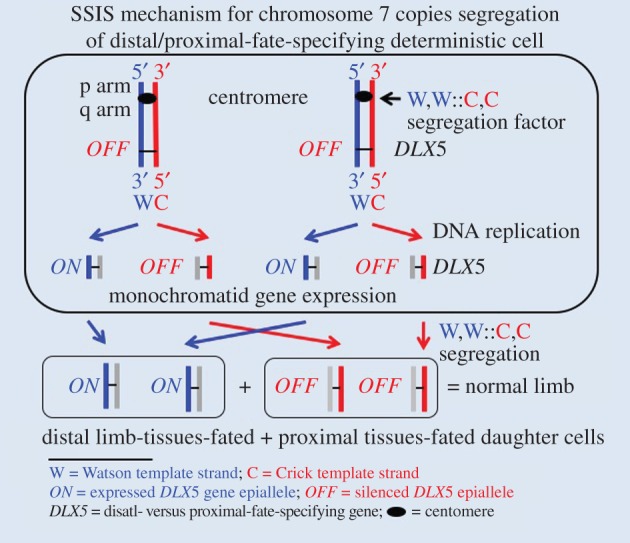
Monochromatid gene expression and selective chromatid segregation components of the hypothetical SSIS mechanism proposed for limb development. The mechanism is advanced for the developmental regulation of the *DLX5* (i.e. *SHFM1*) locus of Chr. 7 in the distal and proximal tissue fates—producing deterministic cell and its daughter cells produced during limb development. The mechanism is based on DNA bases and chemical polarity differences of the arbitrarily designated ‘Watson’ (W = 5′ (top) to 3′ (bottom) chemical polarity) and ‘Crick’ (C = 3′ to 5′) strands of DNA and their inherently asymmetric leading- versus lagging-strand mode of replication for the distal/proximal tissue-specifying developmental *DLX5* gene. The blue lines represent the DNA replication template W strands, the red lines represent the template C strands, and the grey lines represent the strand synthesized in the present replication cycle. For the sake of simplicity, DNA strands are deliberately drawn as straight lines and not as the normally existing double-helix configuration. By this mechanism, designed to achieve monochromatid gene expression, transcriptionally active (*ON*) and silenced (*OFF*) entities of the *DLX5* gene are generated for both Chr. 7 copies in the limb deterministic cell in a DNA strand-specific manner, as drawn here. Accordingly, epigenetically unequal daughter chromosome copies (called chromatids in the G2 phase of the cell) are produced for the *DLX* gene of both Chr. 7 homologue replicas in an ‘*ON/OFF*’ manner owing to a non-canonical strand-specific imprinting process operating in mitotic cells. By our hypothesis, an unknown segregation factor operates at Chr. 7 centromeres to cause selective chromatid segregation by the designated W,W::C,C biased segregation mode drawn in the diagram. The deterministic cell undergoes stem-cell-like asymmetric cell division precisely with respect to adjoining cells that specify tissues of the anterior/posterior (formally equivalent to the left–right body axis) and dorso/ventral axes. To coordinate the development of these three axes, perhaps precise cell-to-cell molecular signalling interactions occur between adjoining cells by maintaining planar cell polarity of respective progenitor cells [38] by oriented cell divisions to produce tissues of the three major axes. As a result, the deterministic cell divides in a way such that both W template strand-containing chromatids, derived from both homologues, are selectively segregated to the distal lineage-destined daughter cell to be placed at the distal location in the developing limb bud, and, consequently, both C template strand-containing chromatids are segregated to the other daughter cell to be placed at the proximal location. A healthy limb develops from descendants of these unequal daughter cells differing in *DLX5* expression. It is not necessarily required that such a patterned gene regulation process is repeated in subsequent cell divisions, so SSIS concerns a single deterministic cell diving in the limb bud. In short, the complementarity of DNA strands leads to generation of alternate and stable *ON* versus *OFF* states of *DLX5* expression on sister chromatids by epigenetic means in the deterministic cell and its immediate daughter cells.

The adjoining *DLX5* and *DLX6* genes are regulated in a strict spatial pattern during embryogenesis. As stated above, these genes are supposedly regulated by the action of both long- and close-range *cis*-acting regulatory elements of the *SHFM1* locus. Interestingly, regulatory elements, such as enhancers, that control *DLX* gene expression in vertebrates are spread in a region of several hundred Kb located both in the 5′- and 3′-flanking regions of the locus. Notably, disrupting the action of these regulatory elements causes limb defects phenotypically similar to those arising from conventional *DLX5* gene coding region mutations [11,16,17,19]. Taken together, ectrodactyly results from the 7q21.1–q22.1 region aberrations, where the dysregulation and/or haploinsufficiency model of *DLX5* is well supported by numerous studies [4,14,18]. Haploinsufficiency is the term used when two copies of the gene are essential for the normal phenotype development. The SSIS mechanism (figure 2) is advanced here to help express *DLX5* from both Chr. 7 homologues in one daughter cell and also to keep both alleles epigenetically silenced in the other daughter cell. This is hereafter named the monochromatid gene expression mechanism proposed to operate in the deterministic progenitor cell [39]. The SSIS mechanism also employs the selective chromatid segregation process such that both template W strand-containing chromatids are segregated to a specific daughter cell whereas both C strand-containing chromatids are segregated to the other daughter cell; this was named the W,W::C,C chromatid segregation phenomenon (figure 2) by referring specifically to those DNA (older) strands that were used as templates for DNA replication in the parental cell [23,36].

After discovering the monochromatid gene expression phenomenon in yeast cells [21,22], we searched for the existence of the selective chromatid segregation phenomenon in biology with the goal of exploiting it for understanding multicellular organisms development. The chromosome-specific selective chromatid segregation phenomenon was first discovered in studies of mouse cells. It concerns a Chr. 7 segregation pattern that changes with cell type [23], and the left–right dynein molecular motor was proposed to act at the centromere to execute the selective chromatid segregation function [24]. It was subsequently discovered that *Drosophila* autosomes usually follow the biased W,W::C,C segregation mode during male germline asymmetric stem-cell division, whereby 50% of stem-cell daughters inherit both template W strands and 50% inherit both template C strands [40]. Moreover, *Drosophila* chromatids carrying the old histones are delivered to the stem-cell daughter and those carrying new histones are delivered to the other daughter cell differentiating into a different cell type [41]. Thus, chromatids of autosomal chromosomes are selectively segregated both by the nature of their W versus C template strands and independently by recognizing the old versus new histones residing on them for this germline asymmetric cell division. Two independent controls therefore operate for the segregation of *Drosophila* autosomes during germline cell mitosis. It is worth noting that precedence exists for the discovery of co-segregation of chromosomes containing ‘immortal’ DNA strands undergoing asymmetric stem-cell division in mouse cells [42,43]. Also, a recent study has unambiguously demonstrated that asymmetric inheritance of template strands in the mouse and human embryonic stem cells occurs at a high frequency when stem cells are induced to differentiate into the three primary germ layers in the embryoid bodies [44]. It may seem unusual, but evidence is accumulating to show that the selective chromatid segregation process in some cases might involve the segregation of single DNA strands to sister nuclei during stem-cell division at critical stages during embryogenesis and cancer development, so named the amitotic metakaryotic cell division [45]. However, in comparison with these examples describing biased segregation of the entire or most of the genome, SSIS concerns biased segregation of one or a set of specific chromosomes to function as an epigenetic mechanism for cellular differentiation. Moreover, different chromosomes might be involved in differentiating cells of different cell types.

The SSIS mechanism is composed of two unrelated phenomena—monochromatid gene expression and of selective chromatid segregation—that function together in mitosis of a specific cell. Although seemingly independent phenomena, the advent of the monochromatid gene expression process had probably provided biological material for the evolution of the selective chromatid segregation phenomenon in diploid organisms. Here, we propose that SSIS operates during mitosis of a limb deterministic progenitor cell to produce one daughter cell that inherits both chromosomally borne transcriptionally expressed *DLX5* copies, whereas the other daughter cell inherits both chromosomally borne, epigenetically silenced *DLX* ‘epialleles’ (figure 2). Such differentiated daughter cells are proposed to activate or repress different sets of target genes in their progeny cells to help develop features specific to each anatomical region of the limb. Currently, it cannot be determined whether the cell with expressed or the one with silenced *DLX* genes activates the distal versus the proximal limb tissue developmental programme. Either case equally supports the SSIS mechanism for precisely controlling the expression versus silencing of the *DLX5* gene at the single-cell level in the limb deterministic cell. Here, we searched the literature to find SHFM1 cases associated with chromosomal aberrations that can be employed to scrutinize validity of the SSIS mechanism proposed here for limb development (figure 2).

## 5. The SHFM translocation t(7q11.21;9p12) aetiology supports the SSIS mechanism for limb development

Although the relevant gene's mutation had not been identified, for many years it appeared that only Chr. 7q21 aberrations cause SHFM1 [5], and only recently have recent reports established that conventional mutations in *DLX5* cause autosomal-dominant SHFM1 [11,16]. Thus, intragenic *DLX5* mutations do cause limb malformations, therefore, the model of *DLX5* gene haploinsufficiency for the SHFM1 development is very well established. Inexplicably, however, most of the syndromic *SHFM1* cases involve chromosomal aberrations, including translocations of the *SHFM1* locus itself or of regions around it. Such translocations within the Chr. 7q21–q22 region exhibit breakpoints that are located hundreds of Kb away from the *DLX* locus, and inexplicably, breakpoints reside both in the centromere-proximal as well as the telomeric side of the locus. As these translocations do not harbour *DLX5* mutations, they were explained by hypothesizing the dissociation of long-range, *cis*-acting regulatory gene controls, such as enhancer sequences, to cause dysregulation of *DLX5* to cause the SHFM1 disorder [4,14,18]. Partially supporting this notion, several *SHFM1* locus proximal sequences were shown to bind to the p63 transcription factor at the *SHFM1* locus. Moreover, haploinsufficiency of the *p63* gene of the *SHFM3* locus likewise causes SHFM [13,14]. Clearly, the precise level of expression of both *p63* and *DLX5* genes is required for healthy limb development.

**Figure 3.**
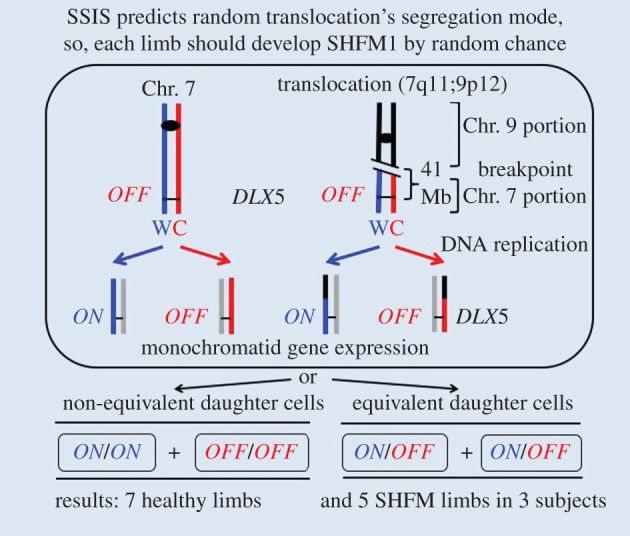
A hypothetical random Chr. 7;9 translocation chromosome's segregation mechanism. Chr. 7 chromatids should follow the usual selective chromatid differentiation and segregation processes, as described in figure 2, but *DLX5* epialleles on translocation chromatids should segregate by the random mode because the Chr. 9 centromere does not follow the selective chromatid segregation process according to our SSIS hypothesis. To help appreciate the segregation mode of different chromosomes, the DNA replication template strands of Chr. 7 are drawn as red and blue lines, while those of the Chr. 9 portion in the translocation chromosome are coloured black. An asymmetric cell division is predicted to produce a healthy limb (bottom left, similar to one described in figure 2), while symmetric cell division should cause SHFM (bottom right). Owing to the random mode of translocation chromosome's segregation, the SSIS mechanism predicts that each limb has a 50% chance of developing malformation (see §5 for details). Results satisfying the prediction have been described [47]. The chromosomal line length is not drawn to scale. All other details are same as those defined in figure 2. Mb, megabase.

As stated before, the model of autosomal-dominant, incomplete penetrance with variable expression of *DLX5* has been proposed in numerous *SHFM1* locus studies, although the authors state the conundrum that the genetic aetiology remains elusive in a substantial proportion of affected individuals because the majority of cases are associated with chromosomal aberrations of the region where subjects harboured the wild-type *DLX5* gene [11,46]. In this situation, interpretation of the existing data has not yet unravelled the mechanism of limb development. We advance here the alternative SSIS mechanism to understand the mechanism of *DLX5* gene regulation likely required for limb development to help to explain the chromosome aberrations aetiology of SHFM1 molecularly (figure 2). To scrutinize validity of the SSIS mechanism, here, we chose specifically to focus on the aetiology of t(7q11.21;9p12) translocation associated with SHFM in which the breakpoint in the centromere-proximal side is located over 41 million bases away from the *DLX5* gene (figure 3) [47,48]. Understanding its aetiology has remained highly enigmatic and we believe its analysis provides a unique window into the mechanism of limb development. In this family, all three family members, a daughter, her father and a grandfather, carry the translocation-developed malformed limbs. Collectively, five limbs of these three members exhibited SHFM and seven limbs did not, while no other family member without translocation-developed malformation. As the disorder occurs rarely (1 in 18 000 births) in the general public, SHFM of all three members in this family is proposed by authors to have been clearly caused by the translocation [47,48]. In addition, it was pointed out that the authors could not exclude the possibility that Chr. 7q11.21 and/or Chr. 9p12 breakpoints might contribute to the SHFM phenotype. As no known *SHFM* locus has been mapped to these regions, such a possibility was suggested by previous workers to be unlikely. Here we advance the alternative SSIS mechanism to explain the biological basis of at least this translocation's aetiology (figure 3).

We speculate here that the translocation interferes with the selective chromatid segregation process of the SSIS mechanism in one-half of limbs because of random segregation operating there (figure 3). As a result, SHFM is predicted to develop when by random chance both developmentally equivalent daughter cells are produced in the limb bud. This anomaly results presumably because the *DLX* gene product level is insufficient owing to the *DLX^ON^/DLX^OFF^* epiallelic constitution and/or both daughter cells have inherited the novel equivalent potential for development; either way, proper development is disrupted. Stated another way, essentially functional haploinsufficiency of *DLX5* is generated in one-half of limbs in a novel way without *DLX5* having been mutated there. Equally likely, when the normally found asymmetric cell division occurs in a limb bud owing to the random mode of chromatid segregation, a healthy limb will develop. This predicted outcome was observed (figure 3). Other translocations with breakpoints lying closer to the *DLX5* gene (but still located hundreds of Kb away from the locus) have been explained by the position-effect dysregulation hypothesis, but the q11 breakpoint of the t(7q11.21;9p12) translocation located over 41 million bases away is unlikely to have caused dysregulation of *DLX5* by the usually invoked *cis*-effects hypothesis. It is this feature of the location of the breakpoint far away from the *DLX5* locus that has the led us to specifically feature this translocation in this communication. Thus, in a novel way SSIS explains all the unexplained features of the autosomal-dominant, incomplete penetrance and haploinsufficiency model previously proposed to explain aetiology of chromosomal aberrations. We surmise that the observation of limbs developing SHFM by random chance while the translocation breakpoint is situated far away from the *DLX* locus provides experimental support for the SSIS mechanism. We propose that the location of the precise translocation breakpoint with respect to the *DLX5* gene is irrelevant to the aetiology and that the observation of random chance of developing SHFM in a limb supports the key feature of the SSIS mechanism positing a single deterministic cell in the limb bud where this mechanism is hypothesized to operate.

In comparison with this translocation causing SHFM in nearly one-half of limbs, three different chain-terminating nonsense mutations in heterozygous condition caused SHFM in 25% (seven out of 24) limbs owing to reduced penetrance and/or unequal sex distribution [11]. These differences in penetrance by translocation and nonsense mutations are not statistically significantly different from each other because of small sample size. Alternatively, this difference may indicate alternative ways in which the disorder develops: one way by making developmentally equivalent sister cells in the case of the translocation and another way by haploinsufficiency in the case of nonsense mutations (figure 3). With the limited data available, it is not yet possible to distinguish between these alternatives.

## 6. The pericentric Chr. 7 inversion aetiology supports the SSIS mechanism for limb development

**Figure 4.**
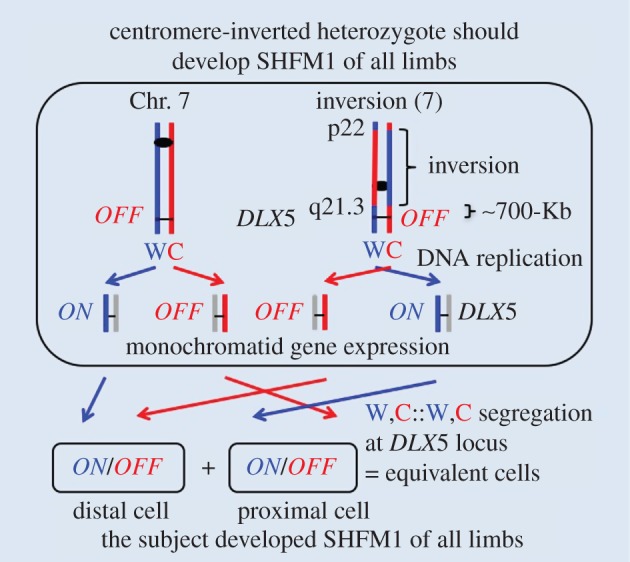
A hypothetical SSIS mechanism proposed for the pericentric-inverted Chr. 7 heterozygous individual. The Chr. 7 should follow the usual selective segregation mode as described in figure 2, but chromatids of the centromeric inverted chromosome should segregate with an opposite orientation to generate the indicated W,C::W,C segregation mode for the *DLX*5 epialleles during mitosis of the deterministic cell. As a result, only developmentally equivalent daughter cells, both containing *ON/OFF* epiallelic constitution, will be produced in each limb: therefore, all limbs are predicted to develop SHFM. Results satisfying the prediction have been described [50]. All other details are the same as those defined in figure 2. The chromosomal line length is not drawn to scale.

Familial rearrangements involving Chr. 7 have been reported very infrequently [49]. SSIS proposes that the orientation of the centromere with respect to the *DLX5* locus in the chromosome is a key component of the mechanism and that the mechanism operates simultaneously on the two Chr. 7 homologues (figure 2). Therefore, an inversion of the Chr. 7 centromere with respect to the *DLX5* locus, when existing in the heterozygous constitution, is predicted to produce symmetric cell divisions in all four limbs deterministic cells. And, as a consequence, SHFM should develop in all limbs of the subject (figure 4). We searched the literature and indeed found such a patient carrying a *de novo* originating Inv(7)p22;q21.3 pericentric inversion associated with all limbs with SHFM [50]. We surmise that the results of this Chr. 7 inversion with one of the inversion breakpoints located in the centromere-proximal side over 700 Kb away from the *DLX5* gene [10] have satisfied a second prediction of the SSIS mechanism; the first one concerns the analysis of a translocation (figure 3). This inversion was previously explained by the hypothesis of *DLX5* gene dysregulation by *cis*-effects of the chromosome rearrangement [10], an explanation similar to those advanced for nearly all other chromosomal aberrations described previously; only some of those aberrations are quoted above.

## 7. A *DLX5*-region inversion supports the SSIS mechanism for limb development

**Figure 5.**
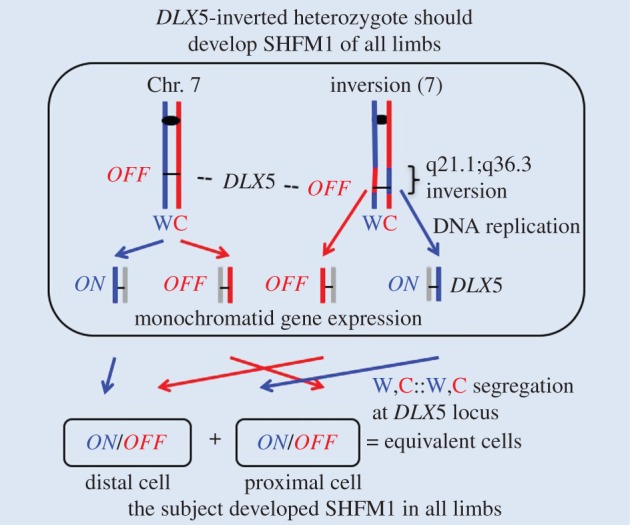
The SSIS mechanism predicts that *DLX5*-region inversion should cause all limbs to develop SHFM. Results satisfying the mechanism have been described in a case report [51] of the inversion drawn in the figure. All other details are the same as those defined in figure 2. The chromosomal line length is not drawn to scale.

A third genetic test of the SSIS mechanism predicts that an inversion of the *DLX5* gene-containing region in the heterozygous constitution should produce symmetric cell divisions in all limb deterministic cells owing to the W,C::W,C selective segregation mode occurring specifically for the *DLX5* locus. And consequently, SHFM should develop in all limbs of the inversion carrier (figure 5). Following a literature search we indeed found a subject containing inv(7)(q21.1;q36.3) paracentric inversion associated with all malformed limbs [51].

## 8. Results of a larger inversion encompassing both centromere and *DLX5* regions of Chr. 7 are consistent with the SSIS mechanism for limb development

A fourth genetic test of the SSIS mechanism predicts that a pericentric inversion that includes the centromere and the *DLX5* locus together in the same inverted segment should not cause SHFM because the normal *DLX* epialleles biased segregation mode will not be altered. Indeed, such a karyotype with Inv(7)p22;q22 pericentric inversion, found in three members of a family, did not cause SHFM [49]; note that *DLX5* is located at q21.3. It is worth pointing out that both of these inversions discussed here have one of their breakpoints located at p22 but only one of them caused SHFM. Although the precise locations of the two p22 breakpoints have not been molecularly defined, observations with two pericentric inversions add weight to the earlier suggestion [10] that a presumptive mutation in the p22 region is unlikely to have caused the disorder by the Inv(7)p22;q21.3 karyotype because no such disorder-causing mutation mapping in this region had been reported previously. Another t(7q22;1p22) translocation did not cause SHFM in four family members [52]; we interpret this observation to mean that the SHFM locus is not located centromere-distal to the Chr. 7q22 region.

Altogether, results of a translocation described in figure 3, two pericentromeric inversions (one described in figure 4) and *DLX5*-region inversion (figure 5) have strongly satisfied unique genetics and developmental biology predictions of the SSIS mechanism. The orientation of the *DLX5* gene relative to the Chr. 7 centromere appears to be essential for proper differentiation of distal-limb structures in humans. We conclude that Chr. 7 centromeric orientation plays a critical role in the selective chromatid segregation hypothesis of the SSIS mechanism, that Chr. 9 centromere supports a random segregation pattern in the limb deterministic cell and that the SHFM locus resides within the Chr. 7q21.3–q22 region: indeed, that is where the *DLX5* locus is situated. Encouraged by four independent corroborations of the SSIS mechanism presented here, we propose that SSIS should be considered as the primary mechanism to define the impact of Chr. 7 aberrations on limb development in future studies, and, perhaps by extension, for understanding the regulatory mechanisms of other developmental control genes in general.

## 9. SHFM caused by the extra copy of the *DLX5/6* region inserted in Chr. 3p21 is consistent with the SSIS mechanism

In humans, malformation-causing mutations act in dominant fashion both through gain or loss of gene function. The SSIS mechanism proposes regulation of the *DLX5* gene such that a precise level of gene product is produced in a specific daughter cell but it is silenced in the other daughter cell (figure 2). Accordingly, the observation of Chr. 7q22–q34 region's insertion into Chr. 3p21 and its association with the three-limb SHFM developmental phenotype of a person [53] are consistent with the SSIS mechanism. Similarly, results of a *de novo* duplication of the 719 Kb region, harbouring only the *DLX5* and *DLX6* genes, found in a patient with SHFM [54] are consistent with the SSIS mechanism. We imagine that such rearrangements affecting gene dosage level might also interfere with the production of the precise level of *DLX5* gene product in the cell.

## 10. Further tests of the SSIS mechanism for limb development

Here we have advanced the SSIS mechanism to explain existing data and to guide future research on limb development. Of necessity, we are obliged to focus on only the final stage of the limb malformation phenotype and its association with specific genetic loci. With this limited knowledge we can only speculate on the details of the SSIS mechanism for limb development. It is not yet possible to identify the hypothesized deterministic limb bud cell, let alone to directly demonstrate the hypothesized monochromatid gene expression and the selective chromosome segregation phenomena operating there (figure 2). Despite these technical limitations, we are pleased with the power of the SSIS mechanism to explain the association of genotype with the phenotype. Additional tests of the SSIS mechanism with predicted developmental outcome should be entertained, some are proposed below, and they should be considered in future research.

As noted above, most cases of SHFM are sporadic in nature and of unknown aetiology [6]. According to the SSIS mechanism proposal, possibly one cause of sporadic cases may be the result of spontaneous, rare somatic recombination events occurring between non-sister chromatids in the genetic interval between the centromere and the *DLX5* locus in the G2 phase of the cell cycle in the deterministic cell (figure 2). A molecular test of this hypothesis predicts the loss of heterozygosity of single-nucleotide polymorphic molecular markers located near the *DLX* gene in tissues of the malformed limb. Also, overall much reduced recombination frequency occurring in mitosis in comparison with that of meiotic recombination frequency may have evolved lest recombination interfere with the operation of the SSIS mechanism of cellular differentiation and development (figure 2).

The mouse equivalent *Dlx5* and *Dlx6* genes mutations cause SHFM, although both copies of both genes must be deleted to generate limb malformation [15,55]. Thus, unlike the situation in humans, haploinsufficiency of these genes does not cause malformation in the mouse. On the other hand, genetic stocks of heterozygous translocations of the *Dlx5/6* loci located on mouse Chr. 6 might provide research material for conducting interesting genetic tests of the SSIS mechanism. For example, such a translocation in the heterozygous condition is predicted to produce developmentally equivalent daughter cells owing to random segregation mode, and SHFM should result in 50% of limbs. This way, one can test whether the precise location of the breakpoint with respect to the *DLX* locus matters and whether only the *DLX* region's orientation with respect to centromeric orientation is relevant. Moreover, translocation homozygous animals are predicted to produce healthy limbs or limbs with unknown phenotype (flipped limbs?). The existing mouse cell lines harbouring site-specific *loxP* recombination sites in the genome [56] could be used to produce the required translocations. Generating mouse stocks with genetically engineered inversions of Chr. 6 centromere or of *Dlx5/6* genes can provide material for additional genetic tests, equivalent to the analysis we presented above for the human Chr. 7 pericentric inversion.

## 11. Should the SSIS mechanism be considered as a general mechanism for development?

Embryonic development requires precise regulation of many developmental genes, likely located in different chromosomes, which are expressed and/or silenced simultaneously and in a developmentally programmed manner. Inappropriate expression of developmental genes in tissues is a major cause of developmental anomalies. Analogous to the analysis presented here for understanding the Chr. 7 aberrations' aetiology, translocations that cause SHFM have provided evidence for the SSIS mechanism operating on Chr. 2 [39]. Notably, three independent Chr. 2q14.1–q14.2 region translocations with breakpoints separated by a relatively large distance of 2.5 million bases, these translocations involving three other chromosomes, were found to be associated with SHFM. Also, the transcriptional regulation of a hypothetical gene located distal to the 2q14.2 breakpoint was speculated to conform to the SSIS mechanism [39]. Supporting this proposal, the *SHFM5* locus located far away from the translocation breakpoints at centromere-distal 2q31.1 region contains the *HOXD*-genes cluster and mutations in this cluster indeed cause SHFM [57]. *HOX* genes, also known as homeotic genes, comprise a functionally related cluster of genes that famously control the embryonic body plan development along the anterior–posterior axis of multicellular organisms. There are four such gene clusters located in different chromosomes in humans. Their encoded homedomain-containing proteins function by regulating the timing and extent of local growth rates of tissues required for patterning during limb and external genitalia development [57]. *HOX* genes function by coordinating the expression of sonic hedgehog, fibroblast growth and other signalling-cascade factors [2]. Interestingly, haploinsufficiency of the *HOXD*-gene cluster [58] causes SHFM in humans, just as we propose here for the *DLX5* gene's functional haploinsufficiency condition generated instead by the Chr. 7 translocation (figure 3) and by a specific centromeric inversion (figure 4). Thus, the diploid dose of human *HOXD* and of the *DLX5* genes is deemed essential for the patterning of limb tissues along the anterior–posterior and/or the distal–proximal axes in humans. Moreover, the haploinsufficiency feature suggests that both alleles of the developmental gene must be expressed simultaneously in a specific cell, just as we propose here for the regulation of the *DLX5* gene though the SSIS mechanism (figure 2).

It was speculated that evolutionary changes in the number of *HOX*-gene clusters and their expression may have caused body-pattern evolution [59]. The order of specific genes in the *HOX* clusters has been very well conserved during evolution from worms to vertebrates, and moreover, homeotic transformation of axial structures results when expression of these genes is altered. Curiously, the order of *HOX* genes in the chromosome is the same as the order of their expression in the anterior–posterior axis of the developing embryo. Reasons for conserving the genes' structural and functional colinearity relationship in evolution are under debate; we propose here that one reason could be for preserving the SSIS mechanism to operate on clustered genes during embryogenesis.

Interestingly and paradoxically, the *HOXD* locus is subject to epigenetic regulation through both gene-repressing Polycomb and gene-activating Trithorax factors. These factors possess histone modifying enzymatic activities to promote active versus inactive chromatin states on gene targets. In mouse ES cells, ‘bivalent domain’ promoters carrying both active and repressive chromatin signatures mark the *HOXD* locus [60]. Note that SSIS requires monochromatid gene expression such that the promoter of the *HOXD* locus is transcriptionally active in one chromatid and epigenetically repressed in its sister chromatid. Thus, the finding of bivalent domain promoters of the *HOXD* locus is in accord to what is predicted by the SSIS mechanism. Moreover, chromatid-specific *mat1* imprinting occurs at a specific sequence of bases on a specific strand and only when it is synthesized by the lagging-strand replication complex in yeast (reviewed in [27]). Therefore, by SSIS, the chromosomal aberrations spanning both the 5′- and the 3′-regions of the *SHFM1* locus might interfere with the function of *DLX* gene regulatory controls by altering the direction of replication of these elements. Thus, the SSIS mechanism provides an alternative explanation to the long-range, *cis*-acting gene regulation controls previously hypothesized to function on both sides of the *HOXD* [61,62] and the *DLX 5* (*SHFM1*) loci [18]. We predict that both Chr. 2 and Chr. 7 undergo the SSIS mechanism in the specific limb cells and that at least one locus that resides centromere-distal to the breakpoints discussed above for either chromosome is required for limb development.

We point out that mechanistic aspects of some of the features of the generic SSIS model are only known from studies with other systems. It is not known which cell is the deterministic cell conforming to the SSIS mechanism in the developing limb (figure 2). We know of only one case, *Caenorhabditis elegans*, where an equivalent deterministic cell is discovered to be the one-celled embryo itself that dictates bilateral left–right neuronal asymmetry to develop many cell divisions later in the adult worm by arguably following the SSIS mechanism [63,64]. For accomplishing development, regulated generation of equivalent and non-equivalent daughter cells at specific stages in development is likely required. By the SSIS mechanism, the process of segregation of W,W::C,C chromatids is proposed to produce non-equivalent daughter cells (figure 2), while the hypothetical W,C::W,C segregation mode (similar to that drawn in figure 4) will produce equivalent daughter cells. One can imagine that mutations in factors required for the specific mode of chromatid segregation will uncover a default segregation mode, which should cause congenital developmental anomalies in mutation carriers. By applying this logic, the SSIS mechanism has been advanced to explain several congenital anomalies that have developed in diverse organisms. These include mouse visceral organs' laterality development, such as *situs inversus* and 50% embryonic lethality of the left–right dynein molecular motor protein mutants [33,34,64], a factor implicated in the Chr. 7 selective chromatid segregation phenomenon that operates during mouse cells mitosis [24]; the generation of bilaterally symmetrical neurons in the *C. elegans* worm by injecting mutant tubulin message into the one-celled embryo [63,64]; human mirror hand movement disorder development owing to *rad51/RAD51* heterozygosity [65]; and the two-coloured wing-spots pigmentation development of the *Bruchus* beetle's *Piebald* gene mutant [66]. Additionally, the mechanism predicts that chromosomal aberrations consisting of translocations or inversions could hinder the distribution of epialleles of developmental control genes to cause congenital developmental anomalies. Similar to the SHFM malformation resulting from Chr. 7 aberrations discussed in this paper and of Chr. 2 aberrations presented above [39], the human psychoses aetiology associated with various translocations with breakpoints covering over 40% of the Chr. 11q arm have been explained by the SSIS mechanism [36,67]. In all these examples, the authors of the studies that first described those disorders stated that the genotype–phenotype relationships have not been understood. We surmise that the SSIS mechanism has explained the molecular basis of their aetiologies very well. Collectively, different features of the SSIS mechanism are evidenced in studies varying from fission yeasts to invertebrates and vertebrates.

Concerning an unrelated issue in humans, approximately 3% to 5% of autosomal genes undergo monoallelic gene expression, and furthermore, they are chosen randomly from maternal and paternal chromosomes by an unknown mechanism [68]. We hypothesize here that the SSIS process, by employing the W,C::W,C biased segregation mode, may provide the mechanism of mammalian monoallelic expression by thus assuring random choice of maternal and paternal autosomal alleles and of genes subject to X-inactivation.

As stated at the beginning of this communication, the morphogen gradient model has been the main paradigm followed for guiding research on biological pattern formation. This model proposes that the fate of a cell is controlled by its position in the developing embryo and by its response to concentration gradients of morphogens with a consistent directional bias laid across the embryo or the developing organ [3]. Morphogen-like mechanisms do operate in limited cases, such as for the morphogenesis of *Dictyostelium* slime mould consisting of cells of only two (prespore and prestalk) cell types [69]. It should be noted, however, that the morphogen model does not readily explain the genetic behaviour of a Chr. 7 translocation discussed here, in which no gene mutation has been implicated for SHFM development and also where SHFM develops in some but not in other limbs of the same patient. The morphogen model of visceral organ laterality development in mice, usually explained by the fluid/morphogen flow of rotating cilia found on cells of embryonic node tissue, has been recently questioned [64] because surprisingly as few as only two cilia located at any place on the entire node are sufficient for laterality development [70]. Moreover, to explain the generation of asymmetric cell division, such as of stem cells, a model proposing the long-range-acting morphogens is unlikely to be the mechanism of cell-fate determination differentially operating on adjoining cells with the precision required. We suspect that the molecules demonstrated to function in cell-to-cell and local, close-range signalling processes might have been misinterpreted as providing convincing support for the morphogen model. Note for example, that the famous Wnt (wingless)/planar cell polarity signalling factor is thought to act as a diffusible morphogen by eliciting long-range-acting cellular controls; shockingly, however, *Drosophila* embryos develop normally after Wnt diffusion is prohibited by tethering the genetically engineered Wnt protein to the membrane of the cell producing it [71]. For the examples discussed above, we had considered the morphogen model but it was considered insufficient for explaining the molecular bases of several congenital disorders enumerated above. Our group [34,64,66] and others [72] have, therefore, questioned the validity of the morphogen model but it continues to be accepted by researchers at large without having been clearly established in any study in our view. It is, therefore, worth pointing out that the original proponent of the morphogen model remarked, ‘Diffusible gradients are out … and that they are too messy’ in a study published in 2009 [73, p. 659].

By realizing these developments, further studies should be designed to test whether an epigenetic SSIS-like mechanism operates in diverse organisms. Concerning the SHFM1 disorder, there is no shortage of molecular and mechanistic studies published in prominent journals for decades, but so far its molecular aetiology has remained undefined. While considering this point, workers in the field would benefit by heeding the advice from Dr James A. Birchler [74], who pointed out that the perception of good research emphasizing only descriptive, mechanistic studies is hindering science because new ideas remain unappreciated. Lacking mechanistic details at the outset, new ideas are considered too risky, unfit both for funding and for publication in prominent journals. The monochromatid differentiation concept, based on DNA strands' chirality for achieving asymmetric gene expression of sister cells, is one such a new idea. Because it has been found to function only in two fission yeasts [27], and due to technical difficulties, it has not been researched whether it operates in any other organism. This might create doubt of SSIS's existence in biology at large because assumptions/details of the SSIS mechanism remain unknown. Concerning limb development, SSIS provides a conceptual framework with which to understand vast amounts of data, a challenge posed by Dr Brenner quoted at the start of this communication [1]. Our thesis should help guide future research on the elusive mechanism of limb development. In sum, evidence summarized here supports our thesis that strand chirality of the double-helix structure of DNA provides the physical basis for mysteries of development during cell proliferation [21,22,27,36,39,65–67], a function that is performed in addition to providing the basis of heredity [28] by following the Mendelian principles of genetics primarily operating in meiosis.
